# Robotisch assistierte und navigierte Pedikelschraubenplatzierung an der subaxialen Halswirbelsäule

**DOI:** 10.1007/s00113-025-01599-2

**Published:** 2025-07-12

**Authors:** Dominik M. Haida, Mike Holl, Oybek Khakimov, Stefan Huber-Wagner

**Affiliations:** 1 Klinikum rechts der Isar, Klinik für Unfallchirurgie, Technische Universität München, Ismaninger Straße 22, 81675 München, Deutschland; 2 Klinik für Unfallchirurgie, Wirbelsäulenchirurgie und Alterstraumatologie, Diak Klinikum Landkreis Schwäbisch Hall, Diakoniestraße 10, 74523 Schwäbisch Hall, Deutschland

**Keywords:** Robotik, Navigation, Hybrid OP, Roboterarm, Cone beam CT (CBCT), Robotics, Navigation, Hybrid OR, Robotic arm, Cone beam CT (CBCT)

## Abstract

**Operationsziel:**

Das Ziel dieses Eingriffes ist es, eine instabile Halswirbelsäulenverletzung zu stabilisieren.

**Indikation:**

Typ-B2-Verletzung nach AOSpine-Klassifikation für Verletzungen der subaxialen Halswirbelsäule und die damit einhergehende Instabilität. Leitlinie und Therapieempfehlungen für diese Verletzung.

**Kontraindikationen:**

Durch robotisch-assistierte und navigierte Techniken ergeben sich keine speziellen Kontraindikationen.

**Operationstechnik:**

Durchgeführt im 3D-Navigations-Hybrid-OP „Robotic Suite“, bestehend aus Navigationseinheit „Curve Navigation System“, robotischem 3D Cone Beam CT (CBCT) „Loop-X“, robotischem Arm „Cirq Arm System“ und Wandmonitor „BUZZ“ (Fa. Brainlab, München, Deutschland).

Die Erläuterung der einzelnen Operationsschritte erfolgt im Video (Englisch), abrufbar auf der Website unter „Supplementary Information“ oder über den QR-Code.

**Operationsschritte:**

Präoperativ: Planungs-CT und Schraubenplanung.

**Intraoperativ:**

Carbontisch, Bauchlage und Mayfield-Klemme. Dorsaler Zugang. Anbringen der Referenzeinheit. 1. CBCT-Scan. Bildfusion. Kontrolle des Fusionsergebnisses. Anfahrt des Roboterarms zum Bohrtrajekt, robotisch assistierte Bohrung. Einbringen des K‑Drahtes. Navigiertes Gewindeschneiden und navigierte Schraubenplatzierung. 2. CBCT Scan, Kontrolle der Schraubenlage. Zufriedenstellende Schraubenlage, Einbringen der Verbindungsstäbe und des Knochenersatzmaterials, steriler Wundverschluss.

**Weiterbehandlung:**

Keine Zervikalorthese. Isometrische Krankengymnastik. Schmerztherapie nach Bedarf und WHO-Schema. Röntgenkontrolle nach 6 und 12 Wochen. Keine Metallentfernung.

**Evidenz:**

Das dargestellte Videomaterial entstammt einer klinischen Routineoperation. Robotisch-assistierte und navigierte Operationen an der subaxialen Halswirbelsäule werden mit guten Operationsergebnissen und hoher Genauigkeiten durchgeführt.

**Video online:**

Die Online-Version dieses Beitrags (10.1007/s00113-025-01599-2) finden Sie das Video zur beschriebenen Operationstechnik (QR-Code unten).

## Abstract (en)

**Objective of surgery:**

The aim of this surgery is to stabilize an unstable cervical spine injury.

**Indications:**

Type B2 injury according to the AOSpine classification for injuries of the subaxial cervical spine and the associated instability. Guidelines and treatment recommendations for this injury.

**Contraindications:**

Robotically assisted and navigated techniques have no special contraindications.

**Surgical technique:**

Performed in the 3D navigation hybrid operating theatre “Robotic Suite” consisting of navigation unit “Curve Navigation System”, robotic 3D cone beam computed tomography (CBCT) “Loop-X”, robotic arm “Cirq Arm System” and wall monitor “BUZZ” (Brainlab, Munich, Germany).

The individual surgical steps are explained in the video (English), available on the website under “Supplementary Information” or via the QR code.

**Surgical steps:**

Preoperative: planning CT and screw planning.

**Intraoperative:**

Carbon table, prone position and Mayfield clamp. Dorsal approach. Attachment of the reference array. 1) CBCT scan. Image fusion. Control of fusion result. Robot arm approach to the drilling trajectory, robotically assisted drilling. Insertion of the K‑wire. Navigated threading and navigated screw placement. 2) CBCT scan, control of the screw positions. Satisfactory screw position, insertion of connecting rods and bone substitute material, sterile wound closure.

**Follow-up:**

No cervical orthosis necessary. Isometric physiotherapy. Adapted pain therapy as needed and according to WHO scheme. X‑ray control after 6 and 12 weeks. No metal removal.

**Evidence:**

The video material shown is from a routine clinical operation. Robotically assisted and navigated operations at the subaxial cervical spine are performed with good surgical results and a high accuracy.

## Hintergrund

Das Einbringen von Pedikelschrauben an der subaxialen Halswirbelsäule stellt sich durch die empfindlichen umliegenden anatomischen Strukturen als sehr anspruchsvoll dar [1, 2]. Allerdings bieten Pedikelschrauben gegenüber anderen Schrauben, wie den Massa-lateralis-Schrauben, Vorteile in Bezug auf die biomechanische Stabilität [3].

Zum sicheren Einbringen von Pedikelschrauben an der Wirbelsäule finden seit einiger Zeit die Navigation und in jüngster Zeit auch die Robotik ihre Verwendung, meist in Kombination mit modernen Bildgebungslösungen [4–9]. Im Bereich der subaxialen Halswirbelsäule ist diese Entwicklung allerdings noch recht jung, da sich noch vor nicht allzu langer Zeit Herausforderungen in Bezug auf die Bildgebungsqualität und ein damit einhergehendes höheres Risiko von Komplikationen gab [1, 2, 4, 5, 7, 8, 10, 11].

## Definitionen und Klassifikationen

Die gängigste und am häufigsten verwendete Klassifikation zur Einteilung von Verletzungen der mittleren und unteren Halswirbelsäule stellt das AOSpine-Klassifikationssystem für subaxiale Verletzungen (C3–C7; Halswirbelkörper 3–Halswirbelkörper 7) der Halswirbelsäule dar [12]. Es wird dabei zwischen Typ-A-, Typ-B-, Typ-BL-, Typ-C- und Typ-F-Verletzungen unterschieden. Verletzungen vom Typ A stellen dabei Kompressionsverletzungen, vom Typ B Zuggurtungsverletzungen, vom Typ BL bilaterale Verletzungen, vom Typ C Translationsverletzungen und vom Typ F Facettenverletzungen dar [12, 13]. Bei den Typ-A-, Typ-B- und Typ-F-Verletzungen wird die Verletzungsmorphologie durch Zahlen weiter differenziert [12, 13]. Läsionen, welche den Typen A4, B und C entsprechen, stellen dabei instabile Verletzungen dar [14, 15]. Dazu werden mit dem Buchstaben *N* der neurologische Status des Patienten und mit dem Buchstaben *M* die Modifikatoren angegeben [12].

## Fallbeschreibung

Der 85 Jahre alte multimorbide Patient dieses Falles wurde unserer Klinik aus einem kleineren Krankenhaus zuverlegt, in welches der Patient nach einem Sturz im häuslichen Umfeld eingeliefert worden war. Bereits am Unfallort wurde die Halswirbelsäule immobilisiert; in unsere Klinik wurde der Patient mit einer Zervikalorthese eingeliefert.

Die dort durchgeführte Bildgebung zeigte als dominierende Befunde eine Distraktionsverletzung der Halswirbelsäule im Bereich C5/C6 (AOSpine-Typ B2:N1) mit Spinalkanalstenose und zusätzlich Frakturen des vorderen und hinteren Beckenrings. Dazu zeigten sich noch weitere Läsionen u. a. im Bereich der thorakalen Wirbelsäule und der Extremitäten. Initiale zeigten sich leichte und transiente neurologische Defizite am rechten Arm (leichtes Absinken im Armhalteversuch) bei einem im Seitenvergleich gering reduzierten Händedruck.

Die Versorgung der Halswirbelsäule erfolgte in 2 Operationen. Bei der ersten Operation erfolgten eine ventral knöcherne Dekompression in mikrochirurgischer Technik (Exoskop), eine ventrale Spondylodese mittels 20-mm-Platte, das Einbringen eines Bandscheibenersatz-Cage sowie das Einbringen von resorbierbarem Knochenersatzmaterial. Bis zur zweiten Operation trug der Patient eine Zervikalorthese.

In der zweiten Operation (dieser Beitrag) erfolgte 9 Tage später eine dorsale Spondylodese von C4–C7 und das Einbringen von keramischem Knochenersatzmaterial.

Des Weiteren wurde anschließend an die Versorgung der Halswirbelsäule, direkt die Versorgung des Beckens mittels SI-S1- und SI-S2-Schrauben (Sakroiliakalschrauben) angeschlossen. Die übrigen Verletzungen wurden konservativ behandelt.

Der Patient konnte am 13. postoperativen Tag nach der hier beschriebenen dorsalen Stabilisierung in eine Akutgeriatrie verlegt werden.

## Operationsindikation

Die Operationsindikation ergab sich aus der Typ-B-Verletzung der Halswirbelsäule und der damit einhergehenden Instabilität der Verletzung, welche auch in Therapieempfehlungen und Leitlinien als Operationsindikation gesehen wird [13, 15].

## Operationstechnik

Diese Operation (Zusatzmaterial online) wurde in unserem 3D-Navigations-Hybrid-OP, der „Robotic Suite“, durchgeführt.

Die „Robotic Suite“ als Hybrid-OP besteht aus der Navigationseinheit „Curve Navigation System“, dem robotischen und fahrbaren 3D Cone Beam CT (CBCT) „Loop-X“, dem Wandmonitor „BUZZ“, dem robotischen Arm „Cirq Arm System“ sowie Mixed-Reality Brillen zur Planung (Fa. Brainlab, München, Deutschland).

Bei dem in diesem Fall verwendeten Schrauben-Stab-System handelt es sich um das Symphony-System der Fa. DePuy Synthes (Umkirch, Deutschland).

Die Schraubenplanung wurde am Tag vor der Operation in einer präoperativen CT-Bildgebung durchgeführt (0:17 min).

Die Lagerung des Patienten erfolgte in Bauchlage, auf einem Carbontisch und mithilfe einer Mayfield-Klemme (0:35 min). Der Kopf zeigt dabei in die Richtung des CBCT.

Nach dem sterilen Abdecken erfolgt der Hautschnitt und die Präparation durch das subkutane Fettgewebe auf die Faszie (0:44 min). Anschließend wird die Faszie längsgespalten. Mit der bipolaren Schere wird in die Tiefe präpariert. Nachfolgend werden die Muskeln bis zur vollständigen Darstellung der Laminae abgelöst. Anschließend wird die Patientenreferenzeinheit auf dem Processus spinosus des Wirbelkörpers Th 1 angebracht (0:51 min).

Nun werden die Vorbereitungen zum ersten Scan durchgeführt. Nach dem Abdecken des Eingriffsbereiches mittels eines Schlitztuches, wird das CBCT eingefahren und ausgerichtet (1:06 min). Der OP-Tisch wird ebenfalls in die entsprechende Position gefahren. Nach der Durchführung der Kollisionsüberprüfung (1:23 min) und dem Anfertigen zweier Feldaufnahmen zur Sicherstellung der Abbildbarkeit des Eingriffsbereiches (1:32 min), wird der eigentliche Scan in Apnoe durchgeführt (1:36 min).

Ist der Scan erfolgt, wird die intraoperative Bildgebung mit der präoperativen Bildgebung, welche die Schraubenplanung enthält, fusioniert (1:47 min). Anschließend findet die Kontrolle des Fusionsergebnis mittels Durchscrollen, Zoom und anhand markanter anatomischer Strukturen statt. Zusätzlich erfolgt eine Verifizierung mittels Aufsetzens des Pointers und das Abgleichen der Position auf dem Bildschirm (2:40 min).

Die nachfolgenden Filmsequenzen zeigen dem Ablauf für die Platzierung der Pedikelschraube C4 rechts.

**Abb. 1:**
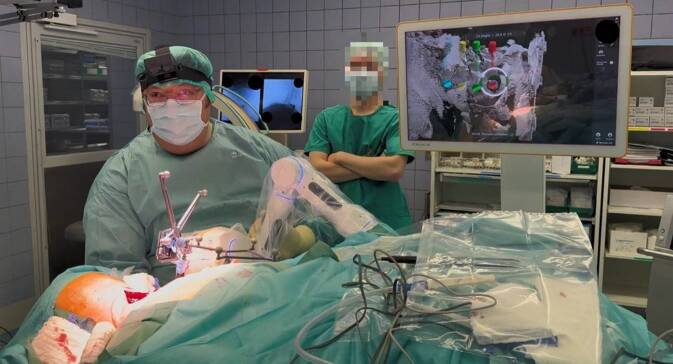
Führen des Roboterarms in die Nähe des geplanten Trajekts

Nach der erfolgreichen Fusion werden die robotisch-assistierten Bohrungen durchgeführt (2:43 min). Als erster Schritt erfolgt die Registrierung der Bohrhülse im System. Daraufhin wird das zu bohrenden Schraubentrajekt ausgewählt. Sobald das passende Trajekt ausgewählt ist, öffnet der Operateur durch das Drücken zweier Knöpfe am Kopf des Armes, alle Gelenke. Unter visueller Kontrolle auf dem Bildschirm führt der Operateur den Roboterarm in die Nähe des zu bohrenden Trajekts (Abb. 1). Leuchtet dieses in grüner Farbe auf dem Bildschirm auf, können die Knöpfe am Kopf des Armes losgelassen werden, woraufhin der Roboterarm eigenständig zur Zielposition fährt. An der Zielposition angekommen, leuchtet der Roboterarm grün. Es wird nun die Bohrhülse in die Carbonführung des Roboterarmes eingeführt. Hierbei empfiehlt sich das Benässen der Führung, um ein möglichst gutes Einbringen der Bohrhülse zu gewährleisten und um möglichst wenig Manipulation auf den Roboterarm auszuüben. Bis zum Wirbelkörper wird die Bohrhülse mittels einer Präparierschere begleitet. Unter visueller Kontrolle auf dem Bildschirm erfolgen die genaue Ausrichtung der Bohrhülse in Linie mit dem geplanten Bohrtrajekt und das Festklopfen der Hülse, um sie stabil in der finalen Borposition auf dem Knochen zu platzieren. Die eigentliche Bohrung wird im Anschluss durchgeführt, an welche sich das Einbringen des Kirschner Drahtes anschließt.

Nun wird der Gewindeschneider im System registriert. Das Gewindeschneiden erfolgt navigiert unter visueller Kontrolle auf dem Bildschirm und mittels Führung des Kirschner-Drahtes (5:19 min). Die Spitze des Gewindeschneiders und damit seine Ausrichtung werden durch eine verlängerte Linie auf dem Bildschirm gezeigt. Diese Linie sollte auf die virtuelle Schraubenspitze auf dem Navigationsbildschirm zeigen, um das Gewinde entsprechend genau in der geplanten Schraubenposition zu schneiden.

**Abb. 2:**
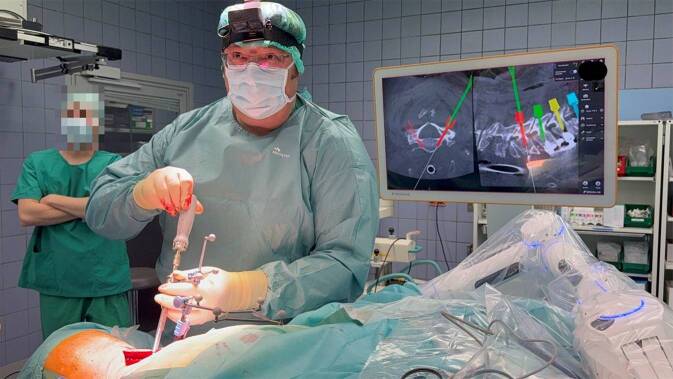
Navigiertes Einschrauben einer Pedikelschraube

Das Eindrehen der Schraube geschieht analog zum Vorgehen beim Gewindeschneiden, ebenfalls navigiert und mittels Führung des Kirschner-Drahtes (6:12 min; Abb. 2). Im Anschluss kann der Kirschner-Draht entfernt werden.

Dieses beschriebene Prozedere wiederholt sich für jede einzubringende Schraube.

Wurden alle Schrauben eingebracht, kann nun der zweite CBCT-Scan durchgeführt werden (7:11 min). Für diesen werden die Tischposition und die Position des CBCT auf die entsprechenden Positionen des ersten Scans angepasst.

Nach dem zweiten Scan wird die Lage aller eingebrachten Schrauben in 3 Ebenen kontrolliert (7:17 min). Die Schrauben werden achsgerecht eingestellt, um die Position zweifelsfrei verifizieren zu können.

Zeigt sich die Schraubenposition zufriedenstellend, werden nun die Verbindungsstäbe eingebracht (8:26 min). In diesem hier gezeigten Fall wurde zusätzlich entschieden, keramisches Knochenersatzmaterial einzubringen. Anschließend erfolgt der Wundverschluss (8:37 min).

**Abb. 3:**
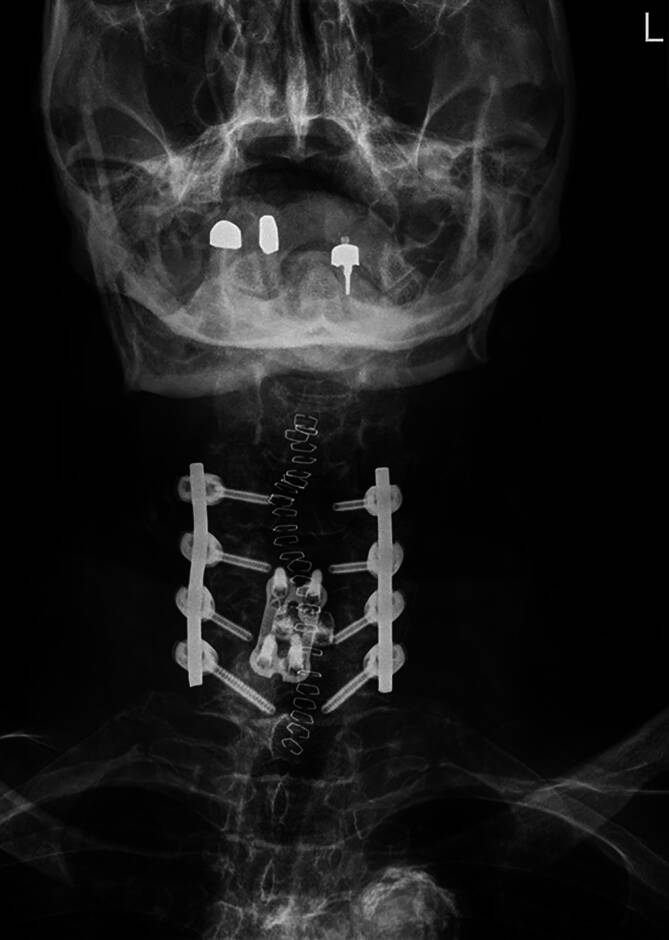
Postoperative Röntgenaufnahme (posterior-anteriore Projektion)

Das Operationsergebnis zeigt sich in der postoperativen Röntgenkontrolle und CT-Bildgebung zufriedenstellend (8:43 min; Abb. 3).

## Postoperative Behandlung

Das Tragen der Zervikalorthese war nach diesem Eingriff nicht mehr notwendig. Es wurde eine isometrische Krankengymnastik angeordnet. Eine adaptierte Schmerztherapie nach Bedarf und WHO-Stufenschema erfolgte. Termine zu Röntgenverlaufskontrollen wurden für 6 und 12 Wochen postoperativ vereinbart. Eine Metallentfernung wird nicht angestrebt.

## Fehler, Gefahren und Komplikationen

Beim Platzieren von Pedikelschrauben an der Halswirbelsäue ergeben sich durch die engen anatomischen Verhältnisse zwei grundlegende Gefahren. So kann eine zu mediale Platzierung der Pedikelschrauben und damit eine Lage im Spinalkanal, zu neurologischen Defiziten bis zur Querschnittslähmung führen. Eine zu laterale Platzierung hingegen, kann die Arteria vertebralis gefährden und damit zu Blutungen und einem Schlaganfall führen.

Um in einem Hybrid-OP generell einen guten und komplikationsfreien Operationsablauf zu gewährleisten und ein gutes Operationsergebnis zu erreichen, sind mehrere Punkte zu beachten.

Vor dem Einsatz eines Hybrid-OP müssen eine gute und detaillierte Instruktion sowie Schulung des beteiligten Personals erfolgen, um menschliche Fehler zu minimieren und einen reibungslosen Operationsablauf zu gewährleisten.

Als wichtigen intraoperativen Schritt sehen wird die Fusion der intraoperativen Bildgebung mit der präoperativen Planung. Dieser Schritt sollte sorgfältig und mit genügend Zeit erfolgen, um ein möglichst gutes und genaues Fusionsergebnis zu erreichen. Wichtig ist, die kritische visuelle und auch haptische Kontrolle des Operationsergebnisses durch den Operateur.

## Evidenz der Technik

Navigierte und robotisch-assistierte Verfahren können in den regulären Klinikbetrieb integriert werden und so zu einem guten Operationsergebnis im Sinne einer genauen Schraubenplatzierung beim Einbringen von Pedikelschrauben an der subaxialen Halswirbelsäule beitragen [4, 5, 8, 16, 17].

Die hier durchgeführte Operation stellt eine Routineoperation in unserer Klinik dar. Seit der Implementierung wurden Schraubenplatzierungen an der kompletten Wirbelsäule, am Becken und an den Extremitäten in diesem 3D-Navigations-Hybrid-OP durchgeführt [4, 9, 16, 18–20 ].

## Fazit für die Praxis

- Das Einbringen von Pedikelschrauben an der Halswirbelsäule ist aufgrund enger anatomischer Verhältnisse sowie empfindlicher Strukturen sehr anspruchsvoll.
- Intraoperativ sind eine akkurate Fusion der Bildgebungen sowie eine Kontrolle des Fusionsergebnisses für eine genaue Schraubenplatzierung essenziell.
- Robotisch-assistiert erfolgt die Bohrung, auf welche das Einbringen des Kirschner-Drahtes folgt.
- Das navigierte Gewindeschneiden sowie das navigierte Eindrehen der Schrauben sollen zusätzlich eine hohe Genauigkeit gewährleisten.
- Das Operationsergebnis kann mittels intraoperativer Cone-Beam-CT-Bildgebung in einer guten Qualität kontrolliert werden.

## Supplementary Information

Video zur beschriebenen Operationstechnik
