# Erratum zu: Robotisch assistierte und navigierte Pedikelschraubenplatzierung an der subaxialen Halswirbelsäule

**DOI:** 10.1007/s00113-025-01631-5

**Published:** 2025-08-12

**Authors:** Dominik M. Haida, Mike Holl, Oybek Khakimov, Stefan Huber-Wagner

**Affiliations:** 1 Klinikum rechts der Isar, Klinik für Unfallchirurgie, Technische Universität München, Ismaninger Straße 22, 81675 München, Deutschland; 2 Klinik für Unfallchirurgie, Wirbelsäulenchirurgie und Alterstraumatologie, Diak Klinikum Landkreis Schwäbisch Hall, Diakoniestraße 10, 74523 Schwäbisch Hall, Deutschland

**Erratum zu:**

**Unfallchirurgie 2025**

10.1007/s00113-025-01599-2

In diesem Artikel wurde der Name des Autors Dominik M. Haida fälschlicherweise als Dominik Marcel Haida angegeben.

Der Originalbeitrag wurde korrigiert.

